# Game Theory and Extremal Optimization for Community Detection in Complex Dynamic Networks

**DOI:** 10.1371/journal.pone.0086891

**Published:** 2014-02-26

**Authors:** Rodica Ioana Lung, Camelia Chira, Anca Andreica

**Affiliations:** 1 Department of Statistics, Forecasting and Mathematics, Babeş-Bolyai University, Cluj Napoca, Romania; 2 Department of Computer Science, Babeş-Bolyai University, Cluj Napoca, Romania; Université de Lausanne, Switzerland

## Abstract

The detection of evolving communities in dynamic complex networks is a challenging problem that recently received attention from the research community. Dynamics clearly add another complexity dimension to the difficult task of community detection. Methods should be able to detect changes in the network structure and produce a set of community structures corresponding to different timestamps and reflecting the evolution in time of network data. We propose a novel approach based on game theory elements and extremal optimization to address dynamic communities detection. Thus, the problem is formulated as a mathematical game in which nodes take the role of players that seek to choose a community that maximizes their profit viewed as a fitness function. Numerical results obtained for both synthetic and real-world networks illustrate the competitive performance of this game theoretical approach.

## Introduction

Networks represent a central model for the description of complex phenomena and they have been studied independently in many different fields such as mathematics, neuroscience, biology, epidemiology, sociology, social-psychology and economy. Recent research trends suggest the emergence of the new science of networks as a field by itself, pioneered by the work of Barabasi [1] and Watts [2]. Typical examples of complex networks in nature and society include metabolic networks, the immune system, the brain, human social networks, communication and transport networks, the Internet and the World Wide Web (WWW). The basic unit of the system is reduced to simple nodes (or vertices) connected by edges (or links) depicting their pairwise relationships. The complexity of real networks is given by non-trivial topological features such as skewed degree distribution, high clustering coefficient and hierarchical structure. Furthermore, local interactions between simple components bring forth a complex global behavior in a non-trivial manner [3]. The most studied features of real-world complex networks include degree distribution, average distance between vertices, network transitivity and community structure [1], [4]-[7]. The focus of the current study is the community structure problem in dynamic complex networks.

In a graph representation of a complex system as a network, nodes with similar properties (or function) have a higher chance to be linked to each other compared to random pairs of nodes. Such nodes tend to form a consistent subgraph (called *community*) highlighted by the dense interconnections. A community in a network can be defined as a group of nodes densely connected with each other but sparsely connected with nodes belonging to other communities [5], [8]. An efficient detection of the community structure can facilitate the identification of functional subunits of the system providing at the same time a powerful tool for the visualization and representation of the network structure. For example, communities may reveal groups of mutual acquaintances in social networks, web pages grouped on the same subject and functional modules in protein interaction networks [7]. Important applications include identifying locations for dedicated mirror servers in order to increase the performance of the WWW, creation of recommendation systems by identifying groups of customers with similar interests, preventing crime by identifying hidden communities on the WWW, vaccination of hubs in the case of developing epidemics and limited vaccinating resources and identifying groups of similar items in social, biochemical and neural networks that can simplify the functional analysis of the networks.

The detection of communities in complex networks is a challenging problem recognized to be NP-hard [9] for which many methods have been proposed in the literature ranging from Community detection methods range from hierarchical clustering [10] (using similarity metrics for the strength of connection between vertices) and divisive algorithms [6], [11] (using the edge betweenness as a weight measure) to random search methods such as evolutionary algorithms [12], [13]. A popular approach to detect communities in complex networks consists in the optimization of *modularity* as a quality function [5], [14]–[17]. Modularity is a measure of the quality for a partitioning proposed by Newman and Girvan [5], [6], [8] that quantifies the deviation of number of interconnections inside a community from the expected density of the same group of nodes in random graphs (with the same expected degree sequence).

An important issue in community detection, less studied however, is the case of *dynamic* communities. This situation is of great significance since most real-world networks change in time and this dynamic behavior should be reflected in the evolution of communities. For example, ad-hoc networks formed by communication nodes constantly change and need to be grouped in order to be able to choose the most efficient communication path. Clearly, the study of dynamic networks can facilitate predictions about the evolution in time of networks from various different areas. Dynamics add another dimension of complexity to the NP-hard problem of detecting communities. An extra mechanism is needed to deal with the network at different timesteps and to include as necessary in the detection of the current community structure, the community structure that existed at the previous timestep.

It should be emphasized that the focus of the current research is on the community detection problem for dynamic networks using online algorithms, i.e. the method must provide a clustering for the network at timestep 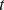 *before seeing the data at timestep* 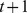. Furthermore, simply using an algorithm to detect communities at different timesteps without considering the evolution of the network is not viewed a good solution as this would be a simple task of community detection repeatedly applied. For instance, methods of information compression proposed in [18], [19] detect communities at different timestamps, without taking into account the structure at a previous timestamp. In contrast, online algorithms should be able to capture the dynamic aspect of network data and adjust online the communities as the network evolves. These features are well described by the concept of *evolutionary clustering* introduced in [20] and engaged in some of the existing methods for the detection of evolving communities [21]–[28]. The strategy is to look for a trade-off between *snapshot quality* (a measure of how good the current community structure is) and *history cost* (a measure of how different the current community structure is compared to the previous one).

The novel approach presented in this paper is based on a game theoretical approach that uses the concept of Nash equilibrium in the following manner: each network node is a player; players have to choose a community; each player has to maximize its payoff computed based on a community score. The Nash equilibrium of this game is a situation in which no node can improve its payoff by unilaterally changing community. When formulating the community detection problem as a game, the existence and uniqueness of the equilibrium depends on the choice of payoff function. Our approach is experimental: an extremal optimization algorithm is used to approximate the Nash equilibrium of the proposed game and its convergence is evaluated by use of numerical experiments performed on synthetic dynamic networks as well as on several real-world complex networks where the dynamic character is captured in the datasets.

## Methods

### Game theory - Prerequisites

Mathematical games model conflicting situations among two or more participants called players. A mathematical game is defined by the triplet formed by the set of players, the strategies available to them and the set of payoff/utility functions for each player. Naturally, all players try to maximize their payoffs. The game is considered non-cooperative if players are not allowed to communicate or interact with each other (i.e. form alliances). Formally a game is defined by 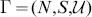 where:

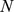
 represents the set of players, 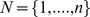, 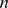 is the number of players;
- for each player 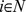, 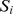 represents the set of actions available to him and 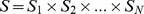 is the set of all possible situations of the game; an element 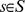 is called a strategy profile, 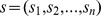, where 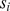 represents the strategy chosen by player 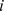 in the profile 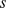;
- for each player 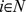, 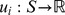 represents the payoff function; 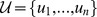.

The ideal situation in which all players can achieve their maximum possible payoff usually does not exist. The most popular solution concept for a non-cooperative game is the Nash equilibrium [29], [30]. A collective strategy 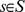 for the game 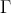 represents a Nash equilibrium if no player has anything to gain by changing only his own strategy.

In [31] the *Nash ascendancy relation* is defined as follows: consider two strategy profiles 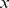 and 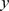 from 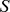. An operator 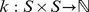 that associates the cardinality of the set composed by the players 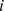 that would benefit if they would change individually their strategy from 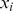 to 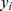.

Let 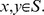 We say the strategy profile 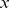 *Nash ascends* the strategy profile 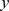 in and we write 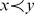 if the inequality 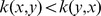



holds.

Thus a strategy profile 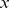 ascends strategy profile 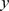 if there are less players that can increase their payoffs by switching their strategy from 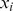 to 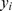 than vice-versa. It can be said that strategy profile 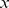 is more stable (closer to equilibrium) then strategy 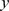.

The strategy profile 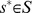 is called non-ascended in Nash sense (NAS) if 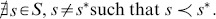



In [31] it is shown that all non-ascended strategies are NE and also all NE are non-ascended strategies. Thus the Nash ascendancy relation can be used to characterize the equilibria of a game. Moreover, this relation can also be used for fitness assignment within heuristic methods such as evolutionary algorithms in order to direct their search towards the Nash equilibrium of a game.

### The Community Detection Game

The community detection problem is considered from a game theoretic point of view by defining the following game:

- **Players:** Consider each *node* of the network as a player; the number of network nodes determines the number of players involved in the game. Let 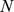 be the number of nodes. The players will be denoted by 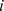, 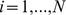;
- **Strategies:** The strategies available to each player are the entire set of communities out of which every node has to choose one (the most suitable for it). A situation of the game is defined as a network cover (community structure) in which each node belongs to a community:

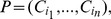



where 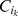 represents the community chosen by player 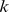;

- **Payoffs** The considered payoff of each player will be the score of the community the player has chosen as defined by Lancichinetti in [32]. This score is computed as the difference between the 'quality' of the community containing that player and the 'quality' of that community without him. The 'quality' of a community is defined as

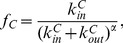
(1)where

– 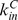 is the internal degree of a community and equals the double of the number of internal links of that community.

– 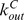 is the external degree and is computed as the number of links joining each member of the module with the rest of the graph.

– 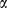 is a positive real-valued parameter, controlling the size of the communities.

The payoff of player 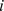 is thus computed: 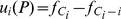
(2)


where 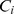 represents the community chosen by player 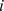 and 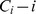 denotes the community 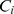 without node 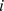. ▴

In this game each player (node) seeks to maximize its payoff by choosing the community that has the most to gain by including it, or has more to loose by not having it as a member.

The Nash equilibrium of this game may be such a situation in which no player (no node) can improve its payoff by unilateral deviation (by changing its community only by himself).

#### The Nash ascendancy relation

can be rephrased as: having two situations 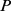 and 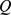 of the game, 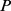 is better than 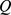 in Nash sense if there are less nodes 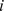 that can improve their payoffs by individually switching from 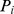 to 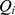 than the players 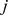 that improve their payoffs from switching from 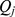 to 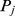.

Thus we compute 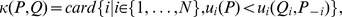



where 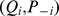 denotes the community structure constructed from 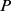 but with node 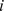 belonging to the community to which it belongs in cover 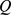.

We say the 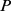 *Nash Ascends* 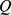 if we have 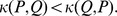 Two strategies (community structures) are indifferent to each other if 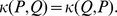


A community structure 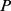 is considered non-ascended (non-dominated) in Nash sense if there does not exists another cover such that 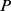 is Nash ascended by it. According to [31] the set of non-ascended strategies coincides with the set of Nash equilibria of the game. A game may have several Nash equilibria which are indifferent to each other from the Nash ascendancy point of view.

The main difference between the game theoretic approach presented here that uses the score from [32] is in the solution concept that is searched for. In [32] the average fitness of the communities is used to evaluate the stability of a cover. The intuition behind our approach is that instead of averaging the fitnesses of all the communities, when simultaneously maximizing all the nodes fitnesses the Nash equilibrium searched for ensures stability against unilateral deviations. Moreover, one of the major challenges in designing optimization approaches for this problem is to propose appropriate fitness functions that highlight ''right'' communities and do not lead to degenerate solutions such as finding a single community containing all nodes. In our approach, by considering that each node has to choose the community that is best suited for him - actually the community to which he contributes the most - and by searching for an equilibrium - optimal/extremal values are avoided and good covers can be found.

### Nash Extremal Optimization for the Dynamic Community Detection Problem (NEO-CDD)

Extremal Optimization (EO) [33], [34] is a general-purpose heuristic for finding high-quality solutions for many hard optimization problems. In this method the value of undesirable variables in a sub-optimal solution are replaced with new, random ones. Within EO a fitness value is assigned to each component of a search vector, the undesired variables are those having the worst fitness.

In the context of games there is a natural fitness assignment between each players strategy and its payoff value as a function of a strategy profile. EO has been successfully applied to Nash equilibria detection for large Cournot games in this manner [35].

For the community detection problem, viewed as the game described above, the NEO-CDD based on Extremal Optimization is proposed. Consider a network of 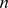 nodes. The main features of NEO-CDD are described in the following.

#### Encoding

Each individual 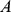 in the population represents a cover over the network represented as an array of 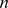 columns and a number of lines corresponding to the maximum expected number of communities denoted by 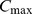. An element 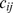 of the matrix is:
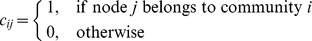



A maximum number of communities that individual 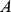 searches for, denoted by 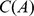, is also assigned, where 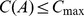.

#### Fitness Assignment

For each node 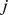 in 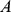 the payoff 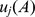 is computed based on equations (1) and (2). A global fitness 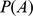 based on the community score is also computed for each cover 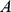.

##### Example

**Figure 1:**
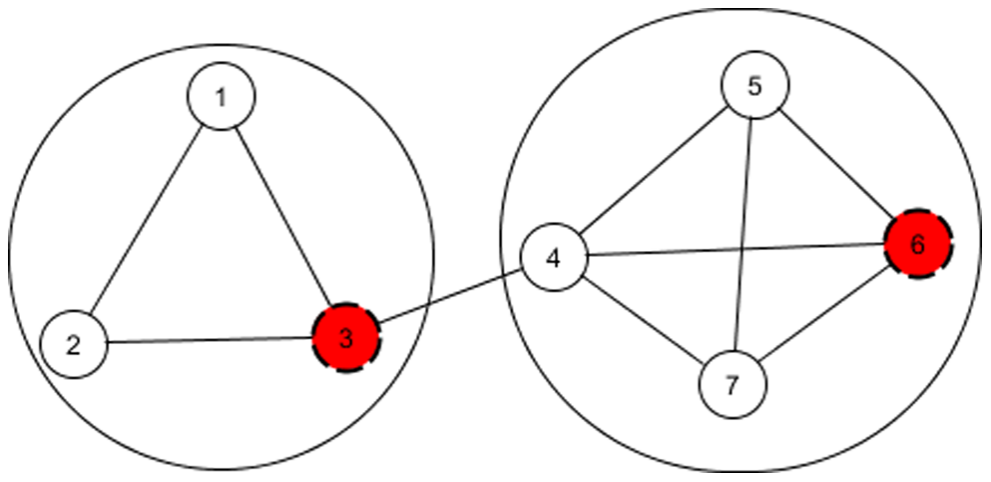
A small network with 7 nodes and 2 communities.

Consider a network with 7 nodes and 2 communities (Figure 1). Table 1 illustrates the encoding of 4 individuals (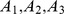 and 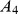) with different number of communities. Columns represent nodes of the network and lines represent communities. The first cover has nodes 3 and 6 (red in Figure 1) in the first community and the rest in the second community. The payoff of the second node from 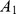 is 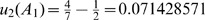.

**Table 1:** 4 individuals encoding covers with 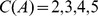.

	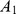	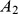	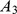	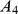
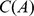	2	3	4	5
C1	0010010	0001000	1010101	0010011
C2	1101101	1100110	0001000	0000100
C3		0010001	0000010	1000000
C4			0100000	0100000
C5				0001000

#### Populations

NEO-CDD evolves a two-leveled population of covers, a parent population 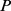 that preserves the most promising solutions and a dummy population 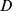 of individuals performing the search following the rules of EO. Both populations have the same size 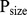. Each individual 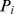 represents the best solution found so far by corresponding individual 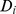 from 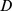.

#### Initialization

At the beginning of the search process all individuals from 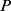 and 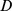 are randomly initialized. For all individuals in 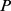 the maximum number of communities searched is set to 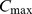. For individuals in 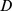 the number of communities searched is set between a minimum number 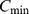 and the maximum 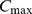. This number is assigned in order from 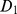 with 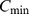 (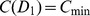), for 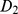 the number is increased with a step 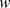 (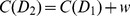) and so on for each 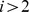 we set 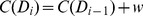 until 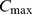 is reached. This process is repeated until all individuals in 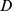 are assigned a community number.

#### Extremal Optimization

Within standard Extremal Optimization two individuals are maintained: one that preserves the best solution found so far and another one that performs the search. NEO-CDD evolves in parallel pairs of individuals from the two populations following the rules of EO: individuals 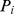 in population 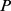 encode the best strategies found by their corresponding 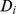 from 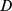.

For each pair of covers 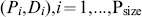, 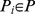 and 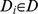 the EO algorithm is applied as described in Table 2 for a number of generations. At each iteration the EO algorithm finds the player (node) from 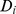 with the worst payoff and randomly generates a new strategy (community) for him. If the new cover Nash ascends 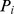 it will replace it and if not - nothing happens. Because this standard EO presents the risk of premature convergence if the player with the worst payoff cannot actually increase it by switching to any other strategy, a parameter 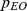 is introduced as the probability to chose a random player to be modified within the EO procedure.

**Table 2:** Nash Extremal Optimization procedure.

1:	**repeat**
2:	For the 'current' configuration 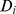 evaluate 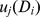 for each player 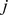 ;
3:	**if** 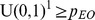 **then**
4:	find the player 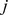 with the "worst payoff";
5:	**else**
6:	randomly generate 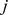 ;
7:	**end if**
8:	change 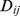 randomly;
9:	**if** ( 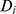 **Nash ascends** 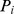 ) **then**
10:	set 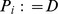 ;
11:	**end if**
12:	
13:	**until** *TerminationCondiyion*;
14:	(Return 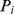 with the best Community Score);

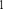
 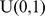 generates an uniform random number between 0 and 1.

At any moment 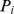 is the best community cover found so far with maximum allowed community number of 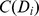.

For a predefined number of communities, the Nash extremal optimization procedure generates correct community structures that are indifferent to each other from the Nash ascendancy point of view. For example, for a network presenting 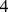 communities, individuals from 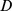 can search for covers containing 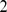 to 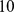 communities, that is 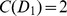, 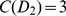, and so on. At some point during the search all individuals will represent valid community structures, with some communities united or divided depending on the maximum number permitted. At the end of a EO procedure an extra-criterion is needed to determine the best community structure detected so the community score [12] (see Appendix S1 for more information) is used.

#### Dealing with Dynamic Aspects

When dealing with dynamic landscapes two major aspects have to be considered: (a) how to determine if a change has occured and then, (b) how to deal with that change.

(a) A change in the network can be easily identified by re-evaluating a sentinel individual at the beginning of each iteration. If its fitness value differs from the previous one, a change has occurred.

(b) When a change is detected NEO-CDD reinitializes all individuals in the 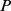 population, keeping population 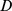 unchanged. In this way the information regarding the previous community structure is available within 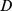 while diversity is induced by individuals in 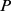.

#### Outline of NEO-CDD

**Figure 2:**
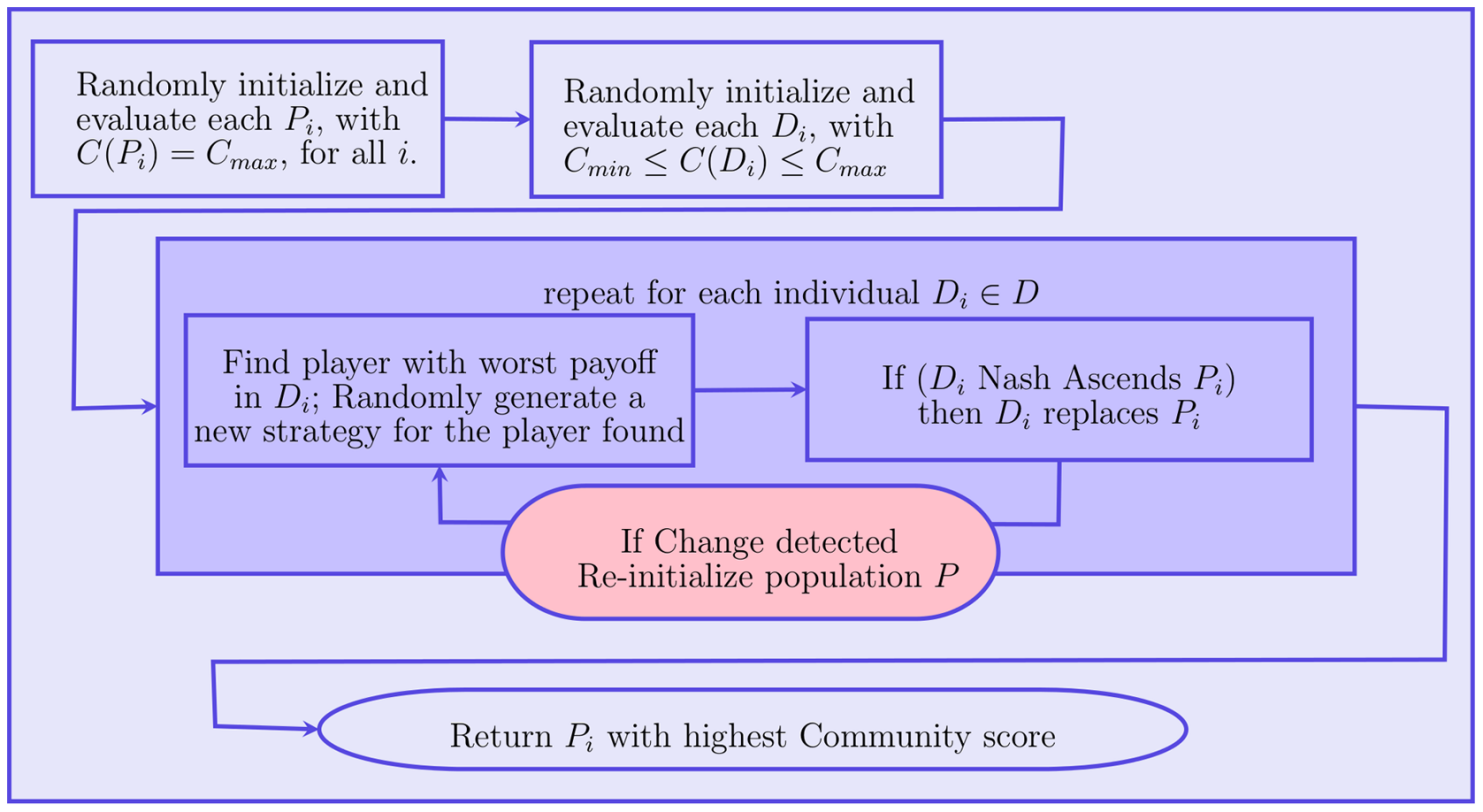
Outline of NEO-CDD.

NEO-CDD evolves the two populations of individuals representing covers for the current network. The first one, 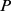, acts as the memory of each individual found by population 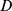 that explores the search space by using a Nash Extremal Optimization procedure. Each time a change is detected in the search space, 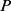 is reinitialized while individuals in 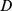 continue their search. Each iteration the individual with the best community score is reported. NEO-CDD is outlined in Table 3. A schematic representation of the method is presented in Figure 2.

**Table 3:** Outline of NEO-CDD.

1:	Randomly initialize 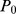 and 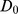 . Set maximum number of communities for all individuals in 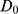 ;
2:	Evaluate 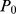 and 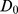 ;
3:	Randomly initialize 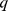 ; Evaluate 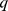 ;
4:	**repeat**
5:	**if** fitness of 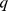 unchanged **then**
6:	Run NEO with 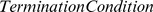 = `fitness of 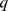 changed` or `maximum number of generations reached';
7:	**else**
8:	Reinitialize 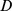 randomly;
9:	**end if**
10:	**until** search complete;

#### Parameters

NEO-CDD uses the following parameters:

- Population size;
- maximum number of generations between changes or number of epochs (necessary to end the search only after the last network change);

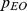
 probability to choose a different node than the one with the worst payoff during EO;
- Initial minimum and maximum number of communities searched 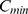 and 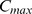 and step 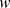;

## Results and Discussion

Computational experiments are performed for both synthetic datasets and real-world complex dynamic networks. This section describes first the network datasets used and then presents the results obtained with their analysis.

### Networks

#### Synthetic Datasets

The synthetic datasets reflect dynamic networks in which edges suffer changes in time and nodes can change their community. The benchmarks are based on the method proposed by Newman [5] for generating network data. The number of nodes in the network is 128 grouped in 4 communities of 32 nodes each. The average degree of each node is set to 16. A number of 50 networks are generated corresponding to 50 timesteps. Dynamics are introduced at each timestep as follows: 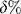 nodes are randomly selected from each community and assigned to the other three communities in a random way. The number of communities stays the same from one timestep to the next. The values considered for 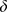 are 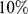 (3 nodes from each community move to the other communities, 1 to each), 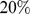 (6 nodes from each community move to the other communities, 2 to each at random), and 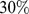 (9 nodes from each community move to the other communities, 3 to each at random).

Edges between nodes of the same community are randomly placed with a higher probability while edges between nodes of different communities are placed with a lower probability. A parameter called 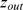 controls the number of links from a node to nodes from other communities. The noise level in the network increases with 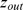. The values used for 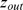 in the current experiments range from 1 to 8 (that is, half of the average degree of a node).

It should be noted that these synthetic datasets are similar to the SYN-FIX benchmark engaged in studies such as [23]–[25]. The network size and community structure is the same, but the number of timesteps considered is only 10 and the number of nodes switching communities every timestep is set to 3 (this corresponds to a 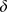 value of 10% in our dataset).

To evaluate the clustering result 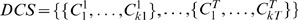, where 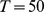, a direct comparison with the known community structure for the network at each timestep 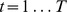 is performed. For this purpose, the *NMI - Normalized Mutual Information* (see Appendix S1 for more information about NMI) is computed to compare the real partition with the detected one. NMI represents a similarity measure between two partitions and is expressed as a real number between 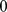 and 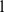 (higher values reflect more accurate partitions). For computing the NMI in our experiments we have used the source code made available by Lancichinetti et al [36] which can be freely downloaded from [37].

#### Football Network

The football data is represented by the games of the National Collegiate Athletic Association (NCAA) Football Division 1-A, collected by James Howell [38]. We selected the years 2005–2009 for the experiments performed in this paper. There are 119 football teams in 2005–2006 and 120 teams starting with 2007. The nodes of the network are represented by the teams, while the edges between nodes represent regular season games between teams. The teams are classified in conferences, each conference containing teams that are playing football games more often with each other than with teams from other conferences. Each conference can therefore be seen as a community, with more intensively connected nodes inside the community and fewer connections between nodes belonging to different communities. There are 12 conferences for the 2005–2009 teams, conferences whose structure slightly changes from one year to another. The dynamism of the communities can therefore be understood as the change that appears in the conferences structure, taking one year as a time step. Because the community structure is known, we use NMI in order to evaluate the algorithm performance.

#### VAST Network

The VAST dataset was part of the 2008 VAST Challenge [39]. It represents the cell phone calls on Isla del Sueno between a selection of 400 persons, over a ten-day period in June 2006. The dataset includes information about the calling phone, receiving phone, date/time, duration and location of the call origination cell tower. We will only use information about the initiator and the recipient of the call, together with the date of the call. We therefore obtain a network where the nodes are represented by the 400 persons, while the edges between nodes represent the cell phone calls between the 400 persons. The dynamism of the communities is given by the changes that occur in the network from one day to another. As the real communities within this network are not actually known the community score and modularity are used in the literature to report the results obtained for this network.

### Results

For all experiments performed numerical results are reported by averaging results obtained over 10 independent runs of NEO-CDD. Whenever possible, if the actual community structure of the network is known, the NMI is used to evaluate and report the results. For the VAST 2008 dataset the community score is reported.

#### Parameter settings

The parameters used by NEO-CDD for each dataset used during numerical experiments are presented in Table 4.

**Table 4:** Parameter settings for NEO-CDD.

Parameter	Synthetic datasets	Football	Vast 2008
Population size	20	30	30
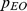	0	0.02	0.02
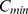	2	8	50
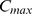	8	16	100
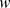	1	1	linearly decreasing from 10 to 1 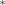

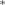
 In order to estimate the value of the optimum number of communities the value of 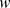 is initially set to 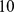 than decreased to 1 linearly while the values of 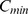 and 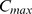 are adjusted based on the community score obtained in the first iterations of the algorithm.

#### Synthetic Datasets

**Figure 3:**
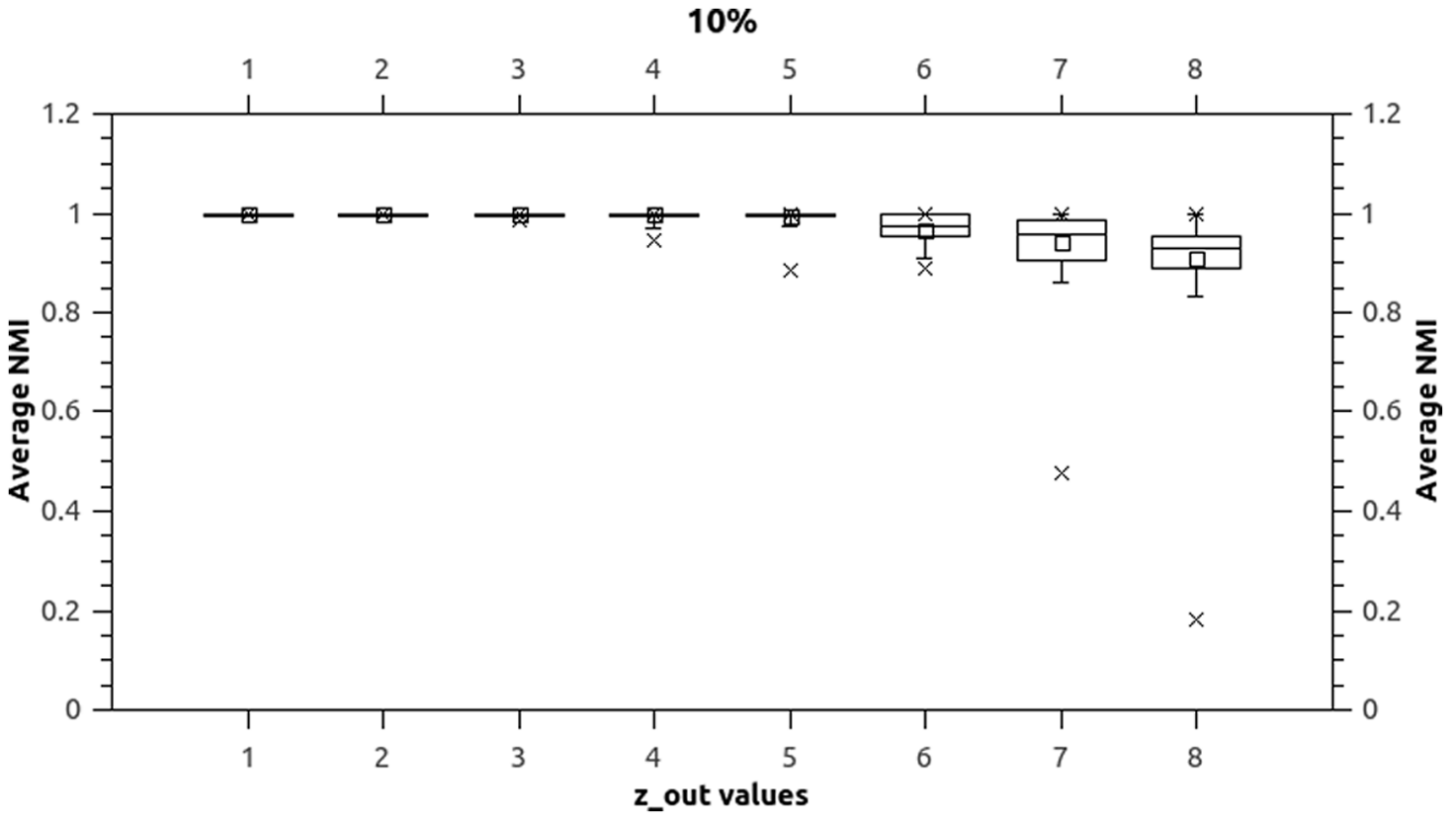
Boxplots (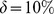). Boxplots indicate that NEO-CDD is capable to detect and maintain the community structures throughout the 50 timestamps with very good NMI values even for 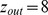.

**Figure 4:**
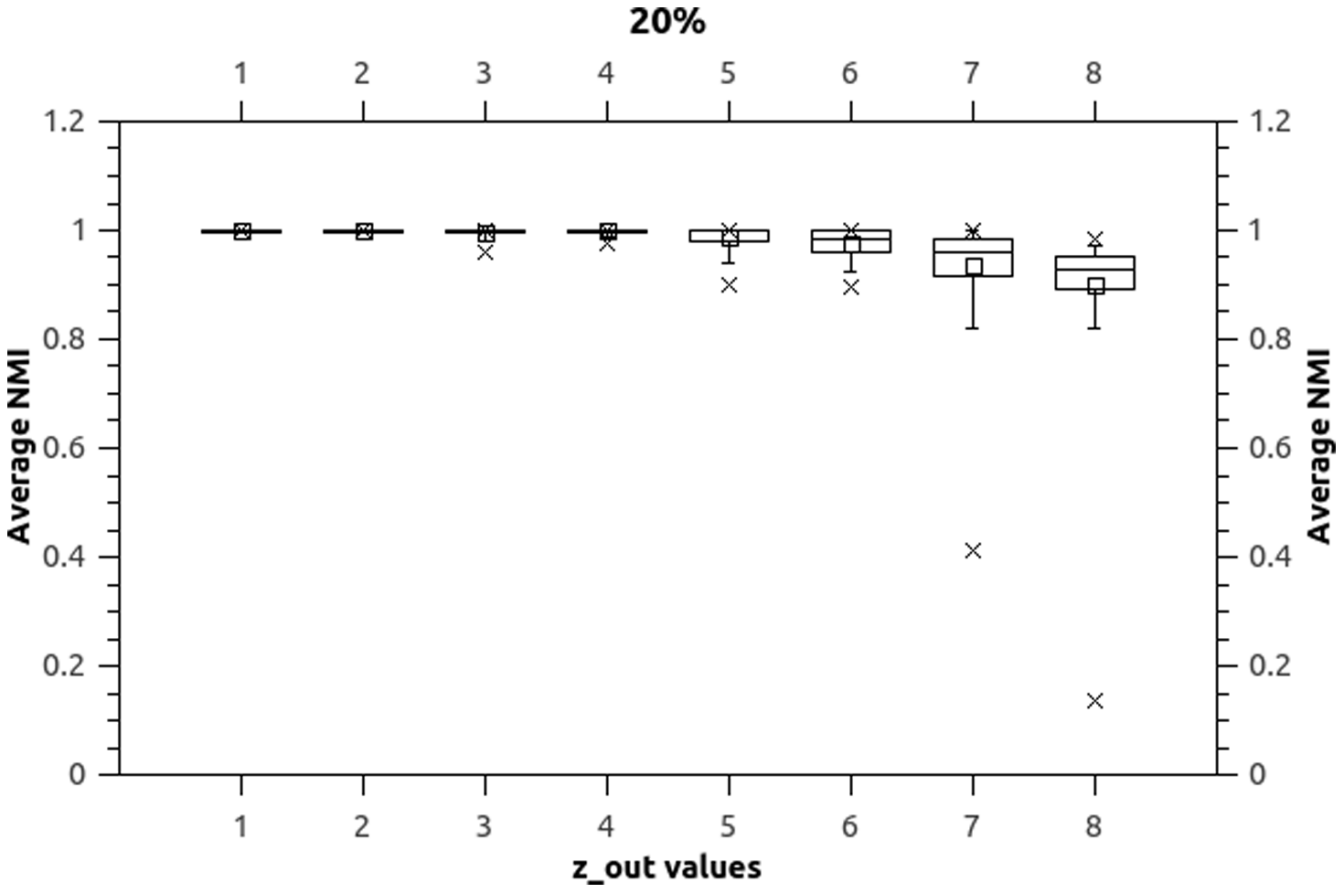
Boxplots (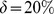). Boxplots indicate that NEO-CDD is capable to detect and maintain the community structures throughout the 50 timestamps with very good NMI values even for 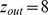.

**Figure 5:**
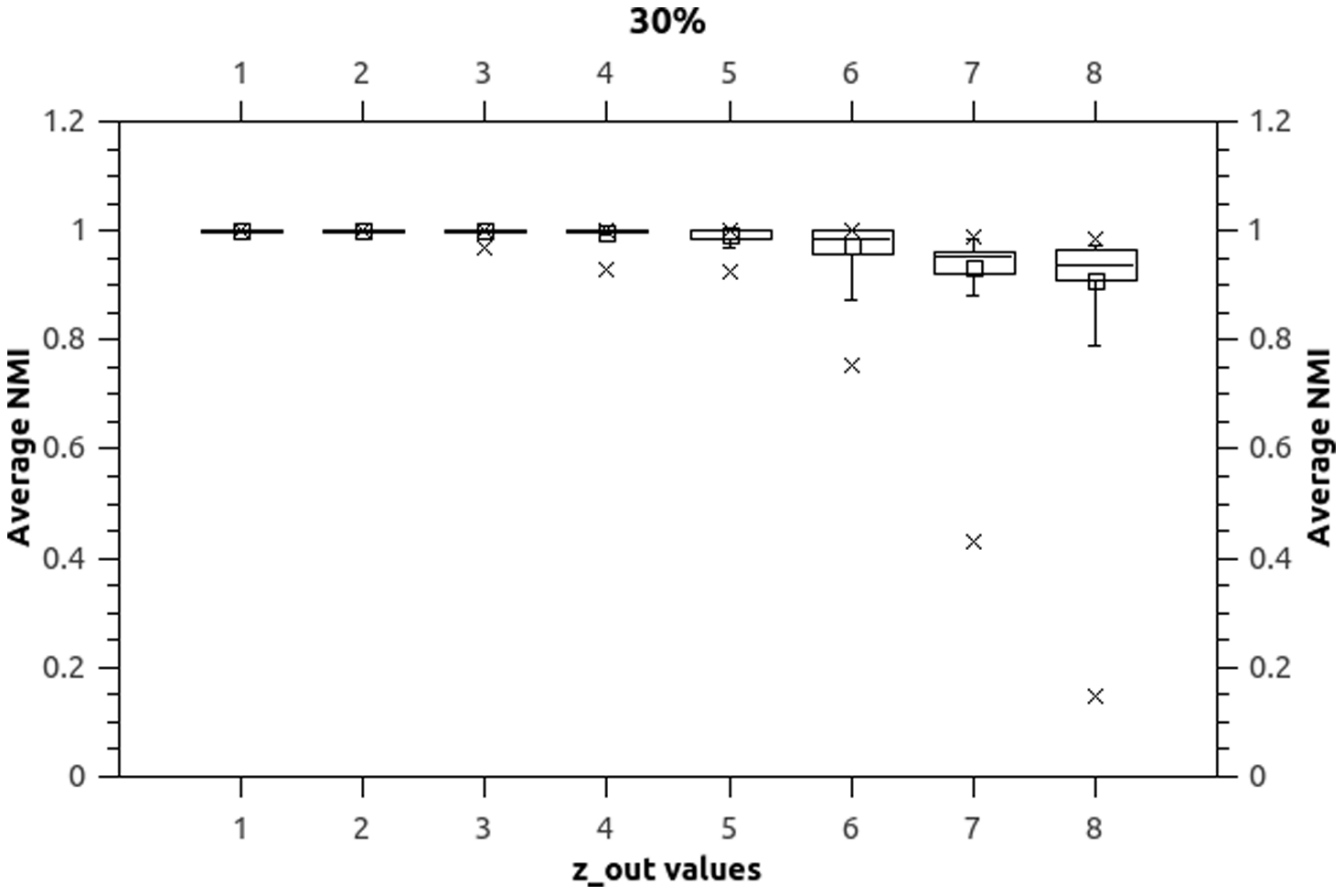
Boxplots (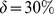). Boxplots indicate that NEO-CDD is capable to detect and maintain the community structures throughout the 50 timestamps with very good NMI values even for 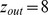.

Both numerical values and box-plots for the average NMI values over the 10 independent runs for the synthetic datasets are presented in Tables 5, 6 and 7 (values 1 and 0 represent the exact results 1 and 0 with no rounding, unnecessary 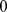 decimal points are omitted) and Figures 3, 4, and 5.

**Table 5:** Descriptive statistics of obtained NMI values for the 10% sets.

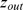	Mean	Std Error	95% CI	Median
			for Mean	
1	1	0	0	1
2	1	0	0	1
3	0.99970	0.00029	5.912e-4	1
4	0.99602	0.00166	0.00335	1
5	0.99327	0.00274	5.510e-03	1
6	0.96748	0.00453	0.00912	0.97372
7	0.93990	0.01124	0.02260	0.95953
8	0.91037	0.01645	0.00632	0.93067

**Table 6:** Descriptive statistics of obtained NMI values for the 20% sets.

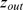	Mean	Std Error	95% CI	Median
			for Mean	
1	1	0	0	1
2	1	0	0	1
3	0.99772	0.00117	2.351e-0	1
4	0.99874	0.00064	1.289e-03	1
5	0.99878	0.00319	6.416e-03	1
6	0.97741	0.00388	7.804e-03	0.98543
7	0.93435	0.01272	0.02557	0.95883
8	0.90078	0.01799	0.03615	0.92606

**Table 7:** Descriptive statistics of obtained NMI values for the 30% sets.

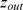	Mean	Std Error	95% CI	Median
			for Mean	
1	1	0	0	1
2	1	0	0	1
3	0.99935	0.00064	0.00129	1
4	0.99734	0.00171	3.454e-03	1
5	0.99273	0.00210	4.228e-03	1
6	0.97107	0.00672	0.01350	0.98548
7	0.93246	0.01113	0.02238	0.95132
8	0.90875	0.01850	0.03717	0.93798

Boxplots represent minimum, median, average, maximum and inter-quartile range for average NMI values over the 50 timestamps for each dataset.

#### Discussion

**Figure 6:**
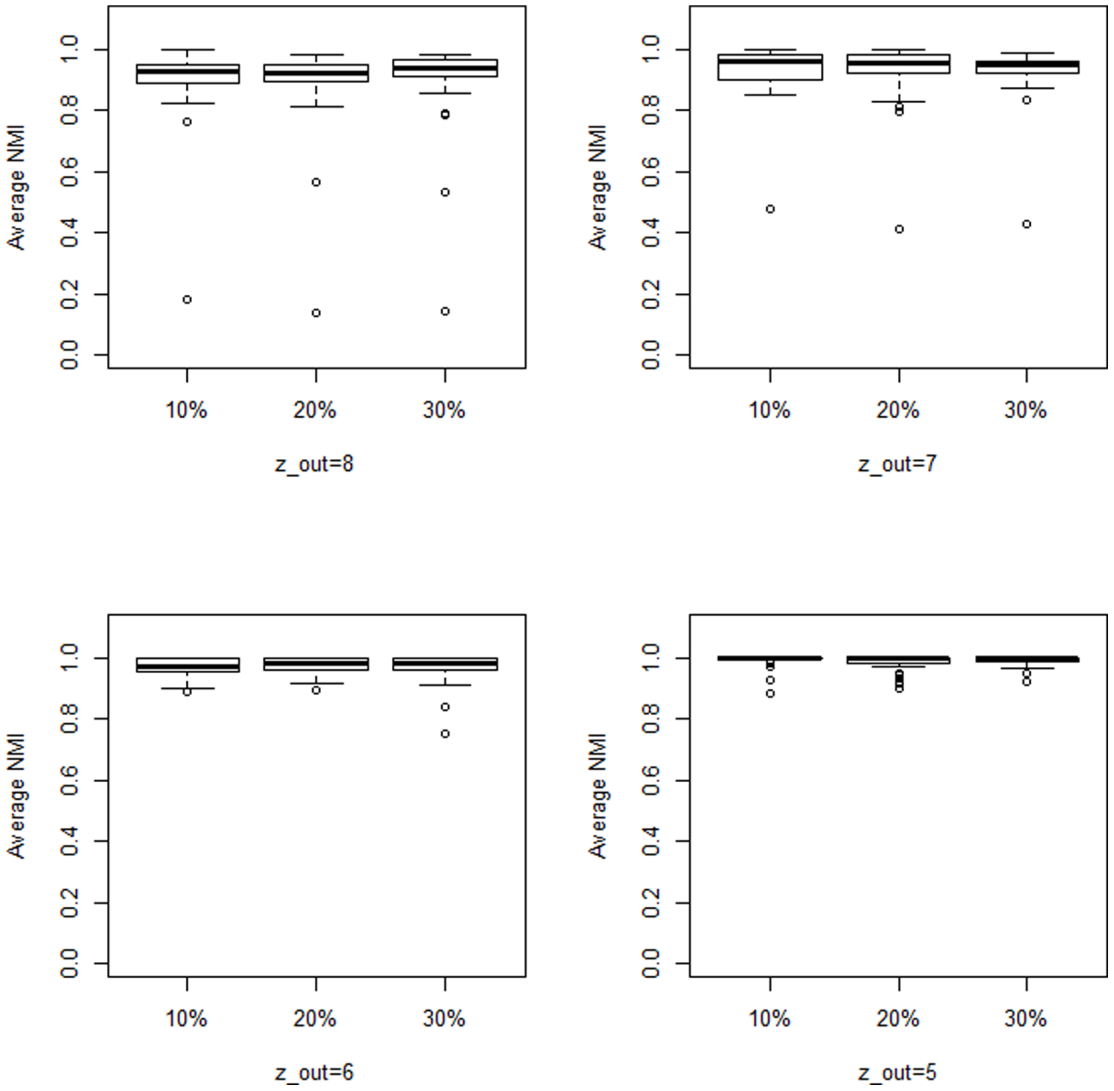
Comparison. Average NMI values obtained for 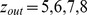. Boxplots indicate that there is no statistical difference between results obtained for 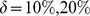 or 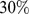.

Figure 6 illustrates the fact that there are no actual differences in behavior when considering different magnitudes of changes within datasets. Wilcoxon sum rank tests performed for all the pairs indicate also that differences between results obtained for different values of 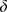 are not significant.

Results obtained for the synthetic datasets for the case 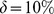 can be compared to the results reported for the SYN-FIX benchmark in [23]–[25]. Indeed, SYN-FIX is created based on the same number of nodes and 3 nodes changing communities each timestep which correspond to the 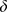 value of 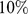 for our synthetic dataset. The difference is that the number of timesteps considered in SYN-FIX is only 10 whereas our dataset contains 50 networks. For 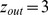, the FacetNet algorithm [23] obtains NMI values ranging from about 0.77 to 0.9 for the 10 timesteps as reported in [24]. For 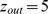, as the number of connecting nodes from other communities is increasing, FacetNet [23] obtains an average NMI value of around 0.2, failing therefore to uncover the community structure. The particle-and-density based evolutionary clustering method presented in [24] obtains similar results with FacetNet for both 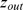 values of 3 and 5. Compared to these two methods, the proposed approach is clearly superior obtaining the maximum NMI value of 1 for 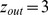 and a very high average NMI of 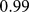 for 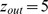 (see Table 5). The DYN-MOGA algorithm [25] is able to trigger better results compared to the methods in [23], [24] reporting an average NMI of almost 1 for 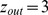 and a NMI above 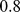 for 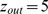. While for small 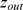 values, DYN-MOGA has a competitive performance, for 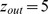 the average NMI reported is considerably lower than that of the proposed model. The DYN-NNIA and DYN-LSNNIA methods [40] report better results compared to DYN-MOGA. For 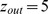 the average NMI is above 0.85 while for 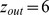 the average NMI ranges between 0.7 and 0.91 for 10 timesteps. Nevertheless, the proposed method reports a higher average NMI (0.97 for 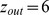) not only for 10% of nodes changing communities each timestep but also for higher 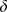 values. The game theoretic approach proposed in this paper clearly outperforms the DYN-MOGA [25] and DYN-NNIA [40] methods as it is able to lead to high NMI values above 0.9 even for high 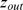 values of 5, 6, 7 and 8, which induce more noise in the dynamic networks.

#### Results for Real-World Networks

**Figure 7:**
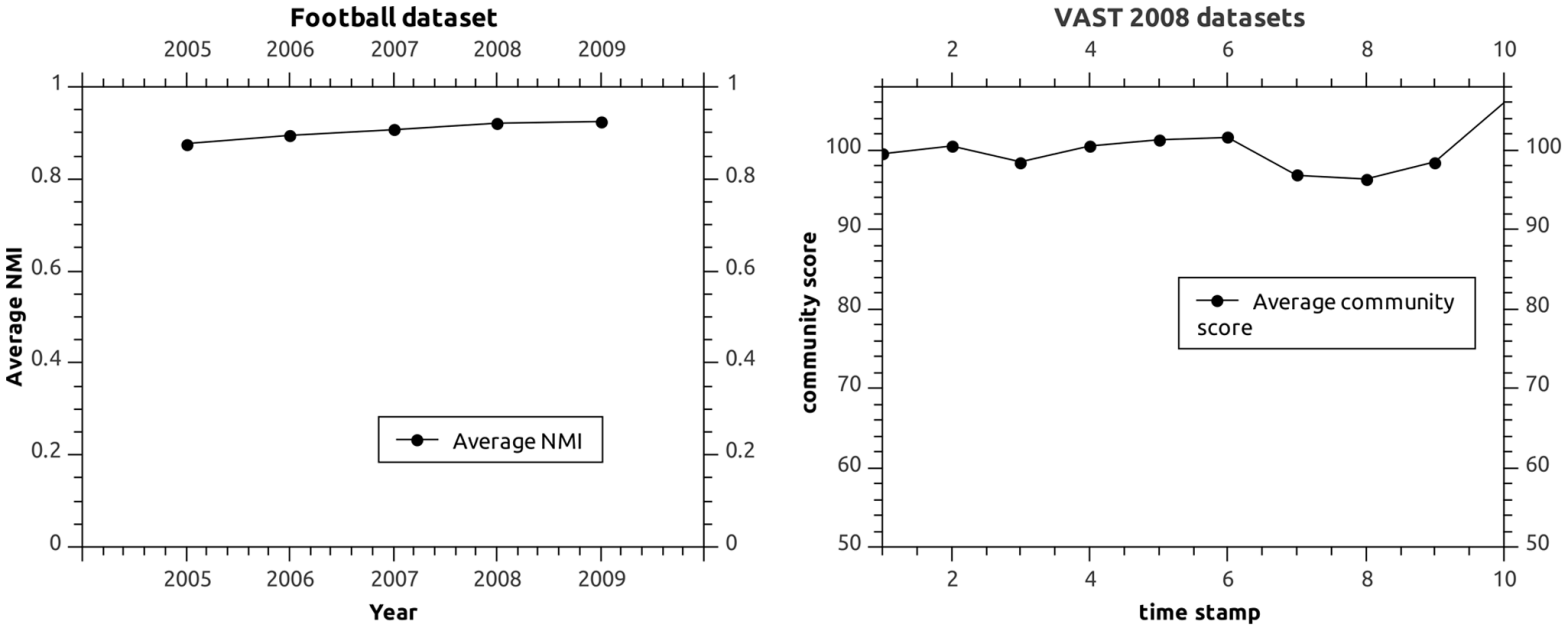
Results obtained for the Football and VAST2008 datasets.

Numerical results obtained by NEO-CDD for the real-world networks are presented in Tables 8 and 9 and illustrated in Figure 7.

**Table 8:** Descriptive statistics of obtained NMI values for the five football datasets.

Year	Mean NMI	St. error	Median	95% CI
				for Mean
2005	0.87661	0.01053	0.86501	0.02382
2006	0.89450	0.00813	0.90986	0.01840
2007	0.90684	0.00780	0.91927	0.01765
2008	0.92098	0.00724	0.93185	0.01638
2009	0.92475	0.00612	0.93127	0.01385

**Table 9:** Numerical results for the VAST2008 challenge dataset (community scores).

Time	Mean Community	St. error	Median	95% CI
stamp	Score			for Mean
1	99.56042	1.28845	98.76910	2.91468
2	100.54726	1.07959	100.70650	2.44221
3	98.55280	0.80641	98.68955	1.82424
4	100.53939	0.81142	99.87540	1.83556
5	101.33094	0.88958	101.01400	2.01239
6	101.62242	0.70801	102.51750	1.60163
7	96.84975	0.71761	97.55800	1.62336
8	96.38221	1.39547	95.64140	3.15677
9	98.54743	0.92559	97.99215	2.09385
10	105.99660	1.15381	106.27450	2.61011

#### Discussion

In [25], the results of DYN-MOGA are given for the Football network in which only the years 2005, 2006 and 2007 are considered to generate the dynamic networks. The average NMI reported by DYN-MOGA [25] is between 0.6 and 0.7 for the three years considered. The corresponding modularity value is around 0.58. As shown in Table 8, the NMI results obtained by NEO-CDD range between 0.876 (for the year 2005) and 0.906 (for the year 2007), which are clearly superior values to DYN-MOGA results reported in [25]. The DYN-NNIA and DYN-LSNNIA methods from [40] improve the DYN-MOGA results for the Football data reporting an average NMI higher than 0.9 for the last four of the five years considered. The approach proposed in the current paper is competitive with the DYN-LSNNIA method as we obtain an average NMI of 0.904 over all five years in the Football network.

For the VAST network, the methods from [25], [40] report an average community score between 92 and 110 [40]. The corresponding modularity values for the covers obtained range between 0.62 and 0.66 [40]. In contrast, the lowest community score obtained by our proposed method is 96.382 at timestamp 8 (see Table 9) while the highest mean community score is around 105. It is known that the structure of the cellphone network changed drastically on the 8th day, which leads to a considerable variation between the community structures from timesteps 7 and 8. As shown in Table 9, our algorithm is able to handle this significant change efficiently as the community score drops from 96.849 at timstep 7 to 96.382 at timestep 8, which is clearly not a major loss of accuracy. On the other hand, the drop in performance reported by DYN-MOGA and DYN-NNIA methods [40] in terms of community score is from around 110 at timestep 7 to just below 100 at timestep 8. This indicates a good reliable behavior of NEO-CDD in handling the changes in network data.

### Final remarks

The proposed game theoretic approach which assigns individual payoffs to each network node provides the framework to efficiently apply the extremal optimization method. By searching for the Nash equilibrium of the game instead of looking for optimal solutions (e.g. Pareto optimal) convergence towards extreme covers (unique community that contains all the nodes /all communities with just one node/etc.) is avoided.

The results obtained by NEO-CDD have been shown to be competitive for both synthetic and real-world dynamic networks. Communities obtained for synthetic networks have a high similarity (shown by NMI) with the known community structure even when the percentage of nodes that change community is as high as 30% and the average internal degree equals the external degree (which creates the most difficult community detection task in a network). For the real-world networks, the ability of NEO-CDD to detect changes in the network data led to good competitive results with clear examples of improved efficiency generated by the proposed approach over existing ones being emphasized in the analysis of the results.

The experimental results confirm the potential of the NEO-CDD approach integrating game theory with extremal optimization in order to address the dynamic complex problem of finding network communities.

## Supporting Information

Appendix S1 (PDF)
