# Supplementary material for: Game Theory and Extremal Optimization for Community Detection in Complex Dynamic Networks
Source: PLoS One. 2014 Feb 26;9(2):e86891. doi: 10.1371/journal.pone.0086891 (PMC3935827; doi:10.1371/journal.pone.0086891)
Supplement: Appendix S1 — (PDF) [file pone.0086891.s001.pdf]

Supporting Information

Game Theory and Extremal Optimization for Community Detection in Complex Dynamic Networks

Rodica Ioana Lung, Camelia Chira, Anca Andreica

Problem Definition

Dynamic networks capture the changes of structure and interconnections over time, which can be traced back in the network structure at different time steps. The network dynamism is generated by nodes/edges additions/removals. Detecting communities in a dynamic network understood in this way can be formalized as follows.

At a certain time step t, the network is given by a set of nodes V^t and a set of links E^t that connect nodes from V^t. A community c of the network at time t is a group of nodes $V_c^t \in V^t$ densely interconnected but sparsely connected with the nodes belonging to $V^t - V_c^t$.

A community structure of the network at time t is given by a set of k communities $C^t = \{C_1^t, \ldots, C_k^t\}$ such that the sets of nodes V_c^t corresponding to each community $c, 1 \leq c \leq k$ at time t represent a partition of V^t:

$$(a) V_c^t \neq \phi, 1 \leq c \leq k,$$

$$(b) \bigcup_{c=1}^{k} V_c^t = V^t,$$

$$(c) V_i^t \cap V_j^t = \phi, 1 \leq i \leq k, 1 \leq j \leq k, i \neq j.$$

A dynamic network DN is a sequence of networks at different time steps $t, 1 \leq t \leq T$, where each network is given by a set of nodes and a set of vertices between these nodes:

$$DN = \{(V^1, E^1), \ldots, (V^T, E^T)\}.$$

Community structure DCS in a dynamic network is a sequence of communities sets at different timesteps $t, 1 \leq t \leq T$, where $\{C_1^t, \ldots, C_k^t\}$ is the community structure of the network at time t:

$$DCS = \{\{C_1^1, \ldots, C_{k1}^1\}, \ldots, \{C_1^T, \ldots, C_{kT}^T\}\}.$$

The final result is therefore a sequence of community structures, one for each timestep.

Evaluation of Community Structures

A problem of great importance in the context of community detection algorithms is defining a good quality measure for the distribution of nodes into communities. The measures described in this section have been traditionally used in the context of community detection in static complex networks.

One of the most well known quality measures is the modularity proposed by Newman and Girvan [1]. Given a division of a network into communities, the modularity is based on the difference between the proportion of edges that connect vertices in a community and the proportion of edges with at least one node in the community (computed at the level of each community). The modularity is denoted by Q and is given in Eq. 1.

$$Q = \sum_{i=1}^{k} (e_{ii} - a_i^2) \tag{1}$$

where k is the number of communities, e is a symmetric matrix of size $k \times k$, each element e_{ij} represents the fraction of edges that connect nodes from community i to nodes in community j (i.e. therefore, e_{ii} is the proportion of edges that connect vertices inside community i) and $a_i = \sum_j e_{ij}$ (i.e. the proportion of edges with at least one node in the community i).

Higher values of the modularity indicate stronger community structures. Modularity optimization has been intensively used in the literature to address the community detection problem [1–5]. However, recent studies indicate that this approach does not necessarily lead to good community structures of networks [6, 7]. The main drawback is the resolution limit of modularity maximization (even with the introduction of a resolution parameter [8]). The maximum value of modularity is essentially unreachable although finding a good approximation of the modularity maximum can be relatively easily achieved by many algorithms. In [6], a comparative analysis of community detection algorithms emphasizes weak results of the modularity optimization approach for benchmark graphs with built-in community structure.

A simple community quality measure has recently been described in [9]. The fitness of a community G is defined by:

$$f_G = \frac{k_{in}^G}{(k_{in}^G + k_{out}^G)^\alpha} \tag{2}$$

where k_{in}^G represents the total internal degree of the nodes in community G, k_{out}^G represents the total external degree and α is a positive real-valued parameter controlling the size of the communities. The fitness of a division P of nodes into communities is the average value of the communities fitness:

$$f_P = \frac{1}{n_c}\sum_{i=1}^{n_c} f_{G_i} \tag{3}$$

where n_c is the number of communities.

The concept of *community score* is proposed in [10] as a quality measure of a partitioning favoring highly intra-connected and sparsely inter-connected communities. Let $M(C)$ (see Eq. 4) be the power mean of community C of order r (where r is a real parameter).

$$M(C) = \frac{1}{|C|}\sum_{i \in C}\left(\frac{k_i^{in}(C)}{|C|}\right)^r \tag{4}$$

The community score CS of a clustering $C_1, \ldots, C_k$ of a network is defined as:

$$CS = \sum_{i=1}^{k} M(C_i) * v_{C_i} \tag{5}$$

where v_{C_i} is the volume of a community C_i defined as the number of edges connecting vertices inside C_i.

The community score has been used with good results as the fitness function in evolutionary approaches to detecting communities in complex networks [10, 11].

Normalized Mutual Information

When the real community structure for a network is known, the *Normalized Mutual Information (NMI)* can be used to assess how close a certain partition solution matches the real known solution.

NMI represents a similarity measure between two partitions and is based on evaluating the Shannon information content of partitions. Let x and y be the cluster labels of a node in two different partitions $\mathcal{X}$ and $\mathcal{Y}$. We assume that cluster labels x and y are the values of two random variable X and Y with joint distribution:

$$P(x,y) = P(X = x, Y = y) = n_{xy}/n \tag{6}$$

where n_{xy} is the number of overlapping nodes between the two clusters labeled by x and y.

The marginal probability distribution of X, respectively Y, is defined as $P(x) = P(X = x) = n_x/n$, respectively $P(y) = P(Y = y) = n_y/n$, where n_x and n_y represent the cluster sizes for labels x and y.

The mutual information $I(X,Y)$ of two random variables X and Y is defined as:

$$I(X,Y) = \sum_x \sum_y P(x,y) \log \frac{P(x,y)}{P(x)P(y)} \tag{7}$$

or

$$I(X,Y) = H(X) + H(Y) - H(X,Y) \tag{8}$$

where H(X) represents the entropy of random variable X associated with a partition and H(X,Y) is the joint entropy.

The mutual information $I(X,Y)$ measures the information that X and Y share (or how much we learn about X if we know Y). For comparing network partitions, $I(X,Y)$ has an important limitation: given a partition $\mathcal{X}$, all partitions derived from $\mathcal{X}$ by further partitioning some of its clusters would have the same mutual information with $\mathcal{X}$. To avoid this problem, NMI has been proposed [4] and is currently extensively used in testing community detection algorithms.

The NMI is defined as follows [4, 6, 7]:

$$NMI(X,Y) = \frac{2I(X,Y)}{H(X) + H(Y)} \tag{9}$$

NMI is expressed as a real number between 0 and 1 with the following significance: a value of 1 for NMI means the two partitions compared are identical whereas a NMI value of 0 suggests that two completely different (independent) partitions are compared.

NMI is useful both in evaluating results obtained for networks with a known community structure as well as measuring the distance between two given network partitions.

Related Methods for Community Detection in Dynamic Networks

The concept of *evolutionary clustering* defined as the problem of producing a sequence of clusterings for timestamped data was introduced by Chakrabarti et al [12]. In this initial problem setting, new data arrives every day and has to be incorporated into an existing clustering. Two different criterias are proposed to evaluate the community structure C^t produced for a particular timestep t: *snapshot quality* and *history cost*. The snapshot quality measures how well the clustering C^t represents the data at timestep t, while the history cost measures the distance between C^t and the clustering C^{t-1} produced for the previous timestep. This framework, known as *temporal smoothness*, assumes that a significant change in the community structure in a very short time is less desirable and therefore *smooths* each community over time under. The temporal smoothness framework is able to catch the evolution of clusters over time and has been used as a starting point in tackling dynamic community structures in many other studies.

Lin et al [13] propose a framework called *FacetNet* for analyzing communities and their evolutions in a unified process. FacetNet uses a stochastic block model for generating communities and probablilistic models for capturing the community evolutions. The KL-divergence between the observed node similarity matrix and an approximate community structure is used to define the snapshot quality. Similarly, the history cost is defined as the difference (computed based on the KL-divergence) between the community structure at time t and the previous structure at time $t-1$. A cost function is defined as a trade-off between the snapshot quality (SQ) and the history cost (HC) as follows: $\alpha \cdot SQ + (1-\alpha) \cdot HC$, where α is

a parameter used to control the emphasis on each part in the total cost. FacetNet follows an optimization process looking to minimize this cost function in order to find the best community structure at a time t. The method proposed by Lin et al [13] is able to explicitly handle dynamic networks but has some limitations related to dealing with a variable number of communities over time and scalability with the network size.

In [14], a particle-and-density based evolutionary clustering method is proposed, which is able to discover a variable number of communities arbitrary forming and dissolving. The concepts of *nano-community* and *l-clique-by-clique* are introduced to capture how dynamic networks evolve over time at a particle level. A community is modeled as a dense subset of nano-communities. The density-based clustering method detects smoothed clusters using a cost embedding technique and optimal modularity. An important feature of this method is that temporal smoothness is applied on the network data and not on the clustering result. Moreover, temporal smoothing is performed both at community and nano-community levels. The cost embedding technique pushes down the cost formula from the cluster level to individual node level. This way, the method is independent from both the similarity measure and clustering algorithm used but still requires a user-defined parameter α to modulate the trade-off between snapshot quality and history cost.

Based on the same temporal smoohness concept, Folino and Pizzuti [11] formulate the problem of community detection in dynamic networks as a multiobjective optimization problem and use genetic algorithms to address it. The mehod, called *DYN-MOGA*, seeks to maximize the snapshot quality while in the same time minimizing the temporal cost. The snapshot quality is measured using the community score CS (see Eq. 5) function [10]. The history cost is assessed based on the NMI (see Eq. 9) between the community structure at the current timestep and the one from the previous timestep. The multiobjective evolutionary model used by DYN-MOGA is the well-known *Nondominated Sorting Genetic Algorithm (NSGA-II)* [15]. An adavantage of this approach refers to eliminating the need of using the α parameter to realize the trade-off between the two conflicting criterias in the temporal smoothness framework. For both synthetic and real-world dynamic networks, DYN-MOGA reports better results compared to both FacetNet [13] and the method proposed by Kim et al [14].

Following the work of Folino and Pizzuti [11], Gong et al [16] develop a multiobjective immune algorithm to solve the community detection problem in dynamic networks. Two objectives are simultaneously optimized: (i) modularity, to measure the quality of community partitions (the snapshot quality) and (ii) NMI as the similarity measure between two consecutive network partitions (the history cost). The multiobjective optimization model used is called *Nondominated Neighbor Immune Algorithm (NNIA)* and is based on a nondominated neighbor-based selection technique, an immune inspired operator and some heuristic search operators. The resulting method for the dynamic problem of communities detection is termed DYN-NNIA. This is further extended in DYN-LSNNIA by integrating a local search mechanism based on the mutation operator. The local search algorithm uses a weighted single objective function to determine the best community label of a node based on the communities of all neighboring nodes. As shown in [16], both DYN-NNIA and DYN-LSNNIA obtain better results compared to DYN-MOGA [11], particularly when the search is augmented with the local search procedure.

References

1. Newman MEJ, Girvan M (2004) Finding and evaluating community structure in networks. Physical Review E 69: 026113+.

2. Guimera AL R (2005) Functional cartography of complex metabolic networks. Nature 433: 895–900.

3. Duch J, Arenas A (2005) Community detection in complex networks using extremal optimization. Phys Rev E 72: 027104.

4. Danon L, Daz-Guilera A, Duch J, Arenas A (2005) Comparing community structure identification. Journal of Statistical Mechanics: Theory and Experiment 2005: P09008.

5. Tasgin M, Bingol H (2006) Community detection in complex networks using genetic algorithm. arXiv .

6. Lancichinetti A, Fortunato S (2009) Community detection algorithms: A comparative analysis. Phys Rev E 80: 056117.

7. Lancichinetti A, Radicchi F, Ramasco JJ, Fortunato S (2011) Finding statistically significant communities in networks. PLoS ONE 6: e18961.

8. Lancichinetti A, Fortunato S (2011) Limits of modularity maximization in community detection. Phys Rev E 84: 066122.

9. Lancichinetti A, Fortunato S, Kertész J (2009) Detecting the overlapping and hierarchical community structure in complex networks. New Journal of Physics 11: 033015+.

10. Pizzuti C (2008) Ga-net: A genetic algorithm for community detection in social networks. In: PPSN. Springer, volume 5199 of *Lecture Notes in Computer Science*, pp. 1081-1090.

11. Folino F, Pizzuti C (2010) A multiobjective and evolutionary clustering method for dynamic networks. In: Proceedings of the 2010 International Conference on Advances in Social Networks Analysis and Mining. Washington, DC, USA: IEEE Computer Society, ASONAM '10, pp. 256–263. doi:10.1109/ASONAM.2010.23. URL `http://dx.doi.org/10.1109/ASONAM.2010.23`.

12. Chakrabarti D, Kumar R, Tomkins A (2006) Evolutionary clustering. In: Proceedings of the 12th ACM SIGKDD international conference on Knowledge discovery and data mining. New York, NY, USA: ACM, KDD '06, pp. 554–560. doi:10.1145/1150402.1150467. URL `http://doi.acm.org/10.1145/1150402.1150467`.

13. Lin YR, Chi Y, Zhu S, Sundaram H, Tseng BL (2008) Facetnet: a framework for analyzing communities and their evolutions in dynamic networks. In: Proceedings of the 17th international conference on World Wide Web. New York, NY, USA: ACM, WWW '08, pp. 685–694. doi: 10.1145/1367497.1367590. URL `http://doi.acm.org/10.1145/1367497.1367590`.

14. Kim MS, Han J (2009) A particle-and-density based evolutionary clustering method for dynamic networks. Proc VLDB Endow 2: 622–633.

15. Srinivas N, Deb K (1994) Muiltiobjective optimization using nondominated sorting in genetic algorithms. Evol Comput 2: 221–248.

16. Gong MG, Zhang LJ, Ma JJ, Jiao LC (2012) Community detection in dynamic social networks based on multiobjective immune algorithm. Journal of Computer Science and Technology 27: 455-467.
